# Repeated mouth rinsing of coffee improves the specific-endurance performance and jump performance of young male futsal players

**DOI:** 10.1080/15502783.2023.2214108

**Published:** 2023-05-16

**Authors:** GHASEM Taheri Karami, MOHAMMAD Hemmatinafar, MARYAM Koushkie Jahromi, JAVAD Nemati, ALIREZA Niknam

**Affiliations:** Department of Sport Science, Faculty of Education and Psychology, Shiraz University, Shiraz, Iran

**Keywords:** Coffee mouth-rinsing, caffeine, explosive power, endurance performance, futsal players

## Abstract

**Background:**

Mouth-rinsing with ergogenic solutions such as carbohydrate and caffeinated drinks has been considered among athletes as a practical nutritional strategy. Therefore, this study aimed to determine the effect of repeated coffee mouth-rinsing (CMR) doses on specific performances of futsal players.

**Method:**

Twenty-four male futsal players randomly participated in this randomized, double-blind, and crossover design study. During the intervention, participants were randomly placed in four different conditions including 1. low-dose CMR (LDC, *n* = 6, ~60 mg caffeine); 2. high-dose CMR (HDC, *n* = 6, ~125 mg caffeine); 3. decaffeinated CMR (PLA, *n* = 6, ~10 mg caffeine); and 4. no CMR (CON, *n* = 6). Vertical jump height was measured at baseline, baseline after CMR (baseline-CMR), immediately after the intermittent futsal endurance test (FIET) (IA-FIET), 5 min after the FIET (5”A-FIET) and 10 min after the FIET (10”A-FIET). Perceived fatigue was also measured by visual analogue scale (VAS) at baseline, IA-FIET, 5”A-FIET, and 10”A-FIET. CMR was also performed at baseline, during FIET (Repeated between levels), and 10’A-FIET. The collected data were analyzed (with SPSS software) by one- and two-way repeated measure ANOVA and Bonferroni post hoc test at *P* < 0.05 level.

**Results:**

The findings of the present study illustrated that the perceived fatigue in IA-FIET increased significantly compared to the baseline which was accompanied by a significant decrease in 5”A-FIET and 10”A-FIET compared to IA-FIET (*P* < 0.05), and no significant difference was observed between conditions in the baseline, IA-FIET, 5”A-FIET, and 10”A-FIET (*P* > 0.05). However, HDC and LDC rose significantly the distance covered in FIET compared to CON and PLA (*P* < 0.05). In addition, HDC increased the FIET performance more than LDC (*P* < 0.05). Although there was no difference between any of the conditions at baseline (*P* > 0.05), baseline-CMR increased significantly the vertical jump height (*P* < 0.05). At IA-FIET, vertical jump height decreased to baseline levels in CMR conditions but increased in 5”A-FIET, which remained constant until 10”A-FIET (*P* < 0.05). In addition, vertical jump height in HDC and LDC conditions was significantly higher than CON in IA-FIET, 5”A-FIET, and 10”A-FIET.

**Conclusion:**

This study showed that repeated CMR with low and high doses is a useful strategy to improve specific futsal performance. However, higher dose CMR appears to have more profound effects on performance improvement than lower doses.

## 1. Introduction

Futsal players, due to the high-intensity nature of the sport, require a high level of fitness for their tactical, technical, and physical demands [1,2]. In other words, high-intensity offensive and defensive actions by each player should be performed in an interval pattern [1]. According to this profile, both anaerobic and aerobic systems are essential in providing the energy required for futsal-specific performance [1]. In addition, endurance in repeated sprints can play a significant role in improving futsal team and individual performance [1]. Therefore, effective nutritional strategies to reduce the perception of fatigue and increase endurance may also be suitable for improving specific futsal performance. One of the strategies to delay fatigue in athletes is to use nutritional compounds before or during exercise [3]. Although industrial nutritional supplements are widely used to reduce fatigue and improve sports performance among athletes [3,4], recently, natural compounds (such as fruits, honey, caffeinated compounds and coffee, red beet juice, etc.) have also been considered to improve the performance of athletes [5]. Coffee is one of the natural ergogenic options for improving sports performance [5–7].

Coffee has a dark color and a bitter taste, and has a stimulating effect on humans due to its caffeine content [5,8]. The amount of caffeine in a coffee drink varies by size, bean origin, roasting method, and other factors, but a typical shot of espresso (1 ounce) typically contains 65 to 85 mg of caffeine [8]. Also, some studies have reported that espresso coffee is an ergogenic aid in athletes [9]. Although the ergogenic effects of coffee are mostly attributed to caffeine but based on the method of coffee preparation, it can include other nutrients such as B-family vitamins, magnesium, and polyphenols that affect sports performance [10–12]. Also, the bitter taste of coffee can affect how the human body reacts [13,14]. The results of some studies show that bitter products can increase performance and also signal to our brain that the organism is ready for action [14]. In addition, the salivary absorption of caffeine [15] has made researchers pay more attention to the effect of mouth-rinsing with liquids containing caffeine on sports performance. Indeed, it has been suggested that the oral cavity has receptors that affect motor performance when stimulated and may increase sports performance by interacting with the nervous system. [13,16]. This mechanism shows why the strategy of rinsing the mouth with certain compounds such as caffeine or carbohydrates improves sports performance [13,16]. Also, the findings from observational studies show that the benefits of rinsing the mouth with carbohydrate and caffeinated drinks are similar to drinking them [13,17]. The practical applications of this approach (Caffeine/Coffee mouth rinsing or CMR) can be significant, as it can improve performance regardless of the digestive discomfort caused by drinking coffee/caffeine [13,18]. However, although there is evidence that caffeine mouth-rinsing leads to improve performance, recently a meta-analysis (2020) showed that the main effect of caffeine mouth rinsing on physical performance is highly heterogeneous [13]. It should be noted that most studies have been conducted on resistance-trained athletes or cyclists, and the findings on team athletes are very limited. Also, these studies have usually used one repetition of caffeine mouth-rinsing, while it seems that repeated caffeine mouth-rinsing can improve the ergogenic effect of this strategy [18]. For example, Dolan et al. (2017) could not show the positive effects of caffeinated beverage mouth rinse (a 10-s mouth rinse) on performance in the Yo-Yo Intermittent Recovery test on collegiate male lacrosse athletes [19]. However, Sinclair and Bottoms (2016) showed that rinsing the mouth with caffeine-containing solutions in a time series (before each round of the arm crank time-trial test, 4 × 25 ml caffeine-containing solutions) improves performance [16]. These findings have been confirmed in another study by Bottoms et al [20]. Concerning power performance, Kizzi et al. (2016) showed that caffeine mouthwash improves power in the conditions of glycogen depletion in trained men [17]. Therefore, the effects of caffeine mouth-rinsing on physical performance require further study, but a potential ergogenic effect may exist in the fasted state and when mouthwash is repeated during exercise [13,17]. Considering the contradictory and limited research on the effects of CMR on athlete’s performance, especially on futsal-specific power and endurance performance, we sought to assess whether the futsal-specific power and endurance performance is affected by CMR in different doses.

## 2. Method

### 2.1. Participants

Twenty-four elite young male futsal players with approximately three years of Iran Futsal League experience (professional futsal players; age: 19.09 ± 1.57 yr; stature: 182.29 ± 9.58 cm; body mass: 70.83 ± 11.46 kg; body mass index [BMI]: 21.19 ± 2.00 kg/m^2^) volunteered to participate in this research. The inclusion criteria included participation in official futsal competitions in the past 3-year, physically healthy with no injuries, no history of allergy to caffeine or coffee, and no history of smoking. Furthermore, exclusion criteria included contracting an infectious disease during the study (cold, flu, etc.), serious musculoskeletal injuries, and taking drugs or ergogenic supplements. Before the implementation of the intervention, the study procedures were explained to the participants, and consent was obtained. This study was reviewed and approved by the Ethics Committee of the Shiraz University, Shiraz, Iran, and carried out under the Declaration of Helsinki.

### 2.2. Sample size calculation

The number of participants in this study was determined based on the study by Silva et al. [21], according to which CMR led to a significant improvement in time to exhaustion compared to placebo (effect size = 0.76). Using G*Power 3.1, considering the confidence interval of 95%, and the analysis power of 0.85, it was found that at least 17 participants are needed for this study. In order to ensure a sufficient sample size, 24 participants were selected for this study.

### 2.3. Study design

**Figure 1.**
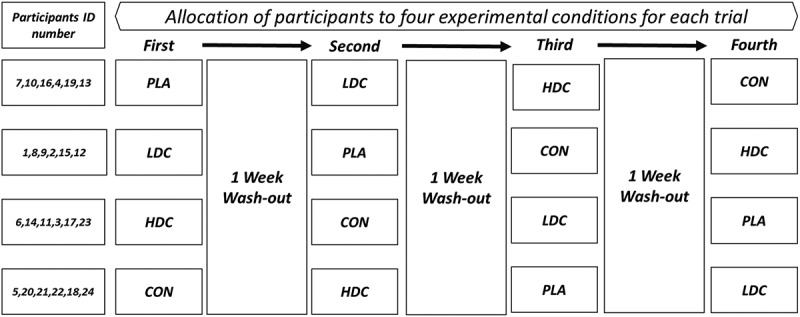
Study design and allocation of participants to four experimental conditions for each trial. LDC: low dose coffee. HDC: High dose coffee, PLA: Placebo, CON: Control.

**Figure 2.**
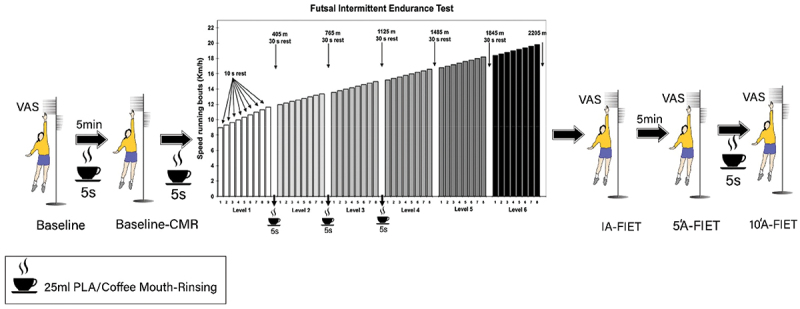
Supplementation and testing protocol FIET. FIET: futsal intermittent endurance test, VAS: visual analogue scale, Baseline-CMR: Baseline CMR, IA-FIET: immediately after the FIET, 5”A-FIET: 5 minutes after the FIET, 10”A-FIET: 10 minutes after the FIET.

This study was carried out in a randomized, cross-over, and double-blinded manner (Figure 1). Before the beginning of the investigation, the participants experienced a familiarization session. During this session, participants were familiarized with all testing protocols and methods. Subjects participated in four separate test sessions. In each test session, the participants were randomly placed in one of the four conditions including 1- high-dose CMR (HDC, *n* = 6), 2- low-dose CMR (LDC, *n* = 6), 3 mouth-rinsing with decaffeinated espresso as a placebo (PLA, *n* = 6)-, and 4 no mouth-rinsing (Control or CON, *n* = 6). Aone-week interval was considered as a washout period for each condition (Figure 1). Each test session included measurement of perceived fatigue by visual analog scale [22,23], vertical jump height, and futsal intermittent endurance test (FIET) (Figure 2). Fatigue perception was measured at rest/baseline, immediately after the FIET (IA-FIET), 5 min after the FIET (5”A-FIET), and 10 min after the FIET (10”A-FIET). Vertical jump height was also measured at baseline, after baseline CMR (baseline-CMR), IA-FIET, 5”A-FIET, and 10”A-FIET (Figure 2). All trials were completed at approximately the same time of day (Between 10 and 11 am). All participants were fed the same breakfast containing 350–400 kcal (64% carbohydrates, 20% protein, and 16% fat) 2 h before the exercise test session. Participants were instructed to maintain their normal diet throughout the testing period, to avoid food and drink in the hour before testing, and to avoid strenuous exercise 24 h before each trial. Participants were provided with a list of dietary sources of caffeine and asked to refrain from consuming these 24 h before each exercise test session. In addition, participants had access to drink water on a self-selected basis during the trials.

### 2.4. Functional tests

The Futsal Intermittent Endurance Test (FIET) is an intermittent fitness test designed to assess the fitness of futsal players, created by Barbero-Alvaréz et al (2005) [24]. The test consisted of running a shuttle over 45 m (3 × 15 m) at progressive speeds dictated by prerecorded audio cues until exhaustion. After each 45 m, participants actively rested for 10 s. Also, after each 8 × 45 m (except after the first level of 9 × 45 m), they rested passively for 30 s before continuing. The increase in speed after 45 m was 0.33 km/h during the first level, and then it was 0.2 km/h for every 45 m. The starting speed was set at 9 km/h [24]. Vertical jump test (Sargent jump) was used to assess the jumping ability of participants, and all assessments in the Sargent jump test were performed three times consecutively (1 min passive rest between them), with the best score recorded as the final result [25].

### 2.5. Dose and preparation of coffees

High-dose espresso coffee (HDC) drink was obtained from the combination of 14 g of caffeinated ground coffee (Lavazza Espresso Italiano, double shot). Placebo (PLA) was obtained from the combination of 14 g of decaffeinated ground coffee (Lavazza Espresso Decaffeinato, double shot). Low-dose espresso coffee (LDC) drink was obtained from the combination of 7 g of caffeinated ground coffee and 7 g of decaffeinated ground coffee. The approximate amount of caffeine in LDC, HDC, and PLA was 60 mg, 125 mg, and 10 mg, respectively. The amount and temperature (180–190 F^o^) of the water, as well as the pressure (9–10 bars: 900–1000 Kpa) applied to prepare coffee, were the same for all. Also, the espresso machine (Gevi 15 Bar 5403) was the same for preparing all coffees.

### 2.6. Coffee Mouth-Rinsing (CMR)

In each test session, participants performed six repeated times of CMR (25 mL each time for 5 s, Figure 2). In each test session, the first VAS and vertical jump height of all participants were measured then they rested for 5 min, and after that, they did the first time CMR (baseline-CMR). Vertical jump height was evaluated five minutes after the first time CMR, and again they had 5 min of rest. The second time CMR was performed immediately before the FIET, and the third, fourth, and fifth times CMR was performed during the rest period after the first, second, and third levels of the FIET. Twice more VAS and vertical jump height were measured immediately and 5 min after the FIET, and then later the sixth time CMR was done. Finally, 10 min after the FIET, the last measurement step of VAS and vertical jump height was performed on participants (Figure 2). It should be noted, coffee cups (along with the instruction label) were prepared for each participant before the tests. The temperature of the coffee was such that it did not burn the mouth (45–50° C). After each CMR, participants emptied the contents of the mouth into the containers placed next to their table. For every three participants, one examiner was responsible for monitoring the CMR.

### 2.7. Training protocol

All participants were members of the same training camp and, their training regime was the same under the supervision of trainers. All subjects participated in the following training program: 5 training sessions of 90 min per week, including 10 min of warm-up, 20 min of physical training (Core stability, Speed, Agility, and Quickness), 10 min of technical training, 20 min of tactical training, 25 min of the training game, and at the end there was cooling for 5 min. Strength and power training occurred once per week as part of team training and consisted of a combination of plyometric (single leg hops, Drop jump, box jump, squat jump: 3 sets × 8 repetitions for each) and resistance exercises (3–4 sets, 10–12 repetitions, 75–80% of a maximum repetition). The type, intensity, load, and duration of training program were similar for all participants. Also one day a week was dedicated to friendly matches, in which each participant played for approximately 10–15 min.

### 2.8. Data analysis

All data were analyzed using descriptive and inferential statistical methods. The data distribution normality was determined using the Shapiro–Wilk test. One-way Repeated measure ANOVA test was used to determine the main effect on FIET performance. A two-way repeated measure ANOVA test (Condition*time) was used to determine the main effect on VAS and vertical jump performance and Bonferroni’s post-hoc test to determine pairwise differences. The data were analyzed by SPSS (version 26, IBM-SPSS Inc., Chicago, IL, USA). The statistical significance level was considered at *p* ≤ 0.05.

### 2.9. Results

Descriptive characteristics (including mean and standard deviation) are reported in Table 1.

**Table 1.** The functional variables (mean ± standard deviation) measured in the four different experimental conditions.

Variables	CON	PLA	LDC	HDC
FIET (m)	1327.5 ± 176.5	1439.8 ± 236.3	1494.4 ± 220	1677.7 ± 206.1
VAS (Baseline) (mm)	7.4 ± 4.6	9.6 ± 5.2	9.2 ± 4.9	8 ± 2.6
VAS (IA-FIET) (mm)	94.2 ± 5.8	90.5 ± 6.7	93.2 ± 5.1	91.5 ± 3.9
VAS (5’A-FIET) (mm)	57.1 ± 9.5	52.3 ± 8.2	54.5 ± 9.7	54.8 ± 6.9
VAS (10’A-FIET) (mm)	39.1 ± 10.8	32.7 ± 10.7	33.6 ± 10.5	34.7 ± 13.6
VJH (Baseline) (cm)	44.5 ± 3.9	44.7 ± 4.1	45.2 ± 4.2	45.1 ± 3.6
VJH (Baseline-CMR) (cm)	44.3 ± 4.2	46.4 ± 4.2	47.4 ± 3.3	48.5 ± 3.2
VJH (IA-FIET) (cm)	41.6 ± 4.3	44 ± 4.2	45.1 ± 4.8	45.8 ± 4.6
VJH (5’A-FIET) (cm)	44 ± 4.5	47.8 ± 5.1	49.6 ± 4.8	50.8 ± 4.4
VJH (10’A-FIET) (cm)	45.2 ± 4.5	48.3 ± 4.1	49.3 ± 4.1	51.1 ± 3.6

Baseline-CMR: Baseline coffee mouth-rinsing, FIET: Futsal intermittent endurance test, IA-FIET: immediately after the FIET, 5”A-FIET: 5 minutes after the FIET, 10”A-FIET: 10 minutes after the FIET, LDC: low dose coffee. HDC: High dose coffee, PLA: Placebo, CON: Control. VAS: visual analogue scale, VJH: vertical height jump.

**Figure 3.**
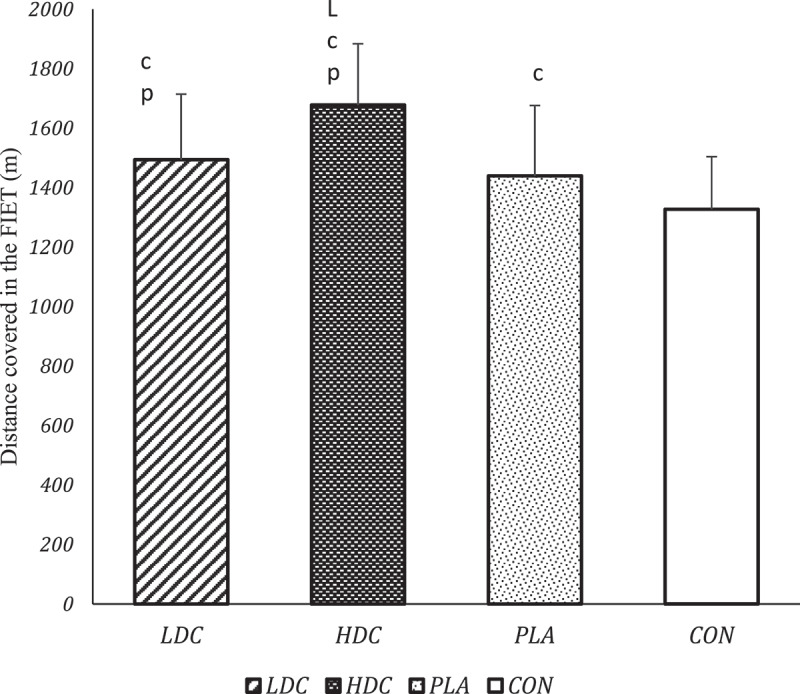
Performance of participants in the FIET. LDC: low dose coffee. HDC: High dose coffee, PLA: Placebo, CON: Control. FIET: Futsal intermittent endurance test. C Significant difference compared to control condition (P < 0.05); L Significant difference compared to LDC (P < 0.05). P Significant difference compared to PLA (P < 0.05).

A significant main effect was observed on FIET performance (F = 60.81, *P* = 0.000, pEta^2^ = 0.72). The FIET performance (Distance covered) was significantly higher in HDC than CON (MD = 350.2, *P* = 0.000, 95% CI [257.8–442.6]), LDC (MD = 183.33, *P* = 0.000, 95% CI [102.3–264.3]), and PLA (MD = 237.9, *P* = 0.000, 95% CI [151.2–324.6]) (Figure 3). Also, the distance covered in the LDC was significantly more than the CON (MD = 166.9, *P* = 0.000, 95% CI [92.1–241.6]) and PLA (MD = 54.6, *P* = 0.005, 95% CI [13.9–95.3]) conditions. In addition, the PLA also increased the distance covered compared to the CON (MD = 112.29, *P* = 0.001, 95% CI [39.8–184.8]) (Figure 3).

**Figure 4.**
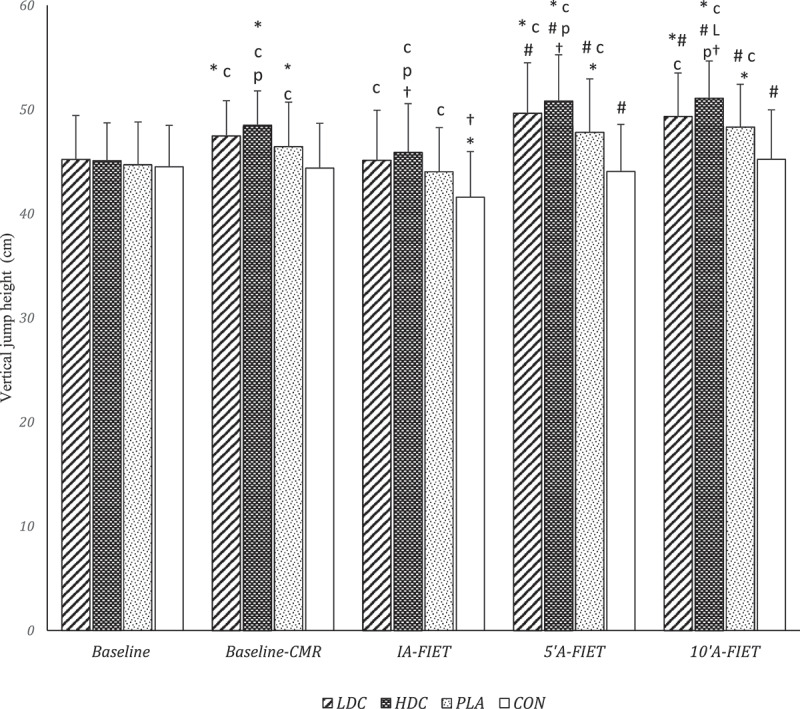
Vertical jump height of participants in different conditions. Baseline: Nutritional intervention has not been done, Baseline-CMR: Baseline coffee mouth-rinsing, FIET: Futsal intermittent endurance test, IA-FIET: immediately after the FIET, 5”A-FIET: 5 minutes after the FIET, 10”A-FIET: 10 minutes after the FIET, LDC: low dose coffee. HDC: High dose coffee, PLA: Placebo, CON: Control. * Significant difference compared to Baseline (P < 0.05)),† Significant difference compared to baseline-CMR (P < 0.05), # Significant difference compared to IA-FIET (P < 0.05 C Significant difference compared to CON (P < 0.05), L Significant difference compared to LDC (P < 0.05). P Significant difference compared to PLA (P < 0.05).

Two-way repeated measure ANOVA showed that the main effect of time [F = 22.7, *P* = 0.000, pEta^2^ = 0.50], condition [F = 48,4, *P* = 0.000, pEta^2^ = 0.67] and interaction (condition × time) [F = 9.16, *P* = 0.000, pEta^2^ = 0.28] on vertical jump height was significant. There was no significant difference between CON and HDC (MD = 0.6, *P* = 0.999, 95% CI [0.2–1.4]), CON and LDC (MD = 0.71, *P* = 0.999, 95% CI [1–2.4]), CON and PLA (MD = 0.18, *P* = 0.999, 95% CI [0.9–1.2]), PLA and HDC (MD = 0.41, *P* = 0.48, 95% CI [0.2–1.1]), PLA and LDC (MD = 0.53, *P* = 0.999, 95% CI [0.8–1.8]), and LDC and HDC (MD = 0.11, *P* = 0.999, 95% CI [1.3–1.5]) in baseline vertical jump performance (Figure 4). The vertical jump height of CON was significantly lower than PLA (MD = 2.05, *P* = 0.001, 95% CI [0.7–3.4]), HDC (MD = 4.11, *P* = 0.000, 95% CI [2.7–5.5]), and LDC (MD = 3.08, *P* = 0.000, 95% CI [1.6–4.5]) after baseline CMR. Also, after baseline CMR, the vertical jump height of HDC was significantly higher than PLA (MD = 2.07, *P* = 0.011, 95% CI [3.7–0.4]), but no significant difference was observed between LDC and PLA (MD = 1.03, *P* = 0.35, 95% CI [0.5–2.5]) or between HDC and LDC (MD = 1.04, *P* = 0.17, 95% CI [2.3–0.2]). Immediately after the FIET, the vertical jump height of CON showed a significant decrease compared to PLA (MD = 2.41, *P* = 0.004, 95% CI [4.2–0.6]), LDC (MD = 3.54, *P* = 0.000, 95% CI [5.20–1.9]) and HDC (MD = 4.3, *P* = 0.000, 95% CI [6–2.6]). However, there was no significant difference in vertical jump performance between PLA and LDC (MD = 1.12, *P* = 0.25, 95% CI [0.4–2.6]) and between LDC and HDC (MD = 0.75, *P* = 0.13, 95% CI [0.1–1.6]) at IA-FIET, but HDC had a higher vertical jump compared to PLA (MD = 1.9, *P* = 0.038, 95% CI [0.1–3.7]). Five minutes after the FIET, the vertical jump height of PLA (MD = 3.75, *P* = 0.000, 95% CI [1.7–5.7]), LDC (MD = 5.59, *P* = 0.000, 95% CI [3.4–7.80]) and HDC (MD = 6.77, *P* = 0.000, 95% CI [4.9–8.7]) increased significantly compared to CON. There was no significant difference in vertical jump height between PLA and LDC (MD = 1.84, *P* = 0.06, 95% CI [0.1–3.7]) and between LDC and HDC (MD = 1.18, *P* = 0.11, 95% CI [0.1–2.5]) at 5”A-FIET, but compared to PLA, the vertical jump height of HDC was higher (MD = 3.02, *P* = 0.000, 95% CI [1.5–4.5]). The vertical jump height of PLA (MD = 3.07, *P* = 0.005, 95% CI [0.8–5.4]), LDC (MD = 4.11, *P* = 0.000, 95% CI [1.8–6.5]), and HDC (MD = 5.85, *P* = 0.000, 95% CI [3.5–8.2]) were significantly increased compared to CON at 10”A-FIET. There was no significant difference between PLA and LDC (MD = 1.04, *P* = 0.42, 95% CI [0.5–2.6]) in vertical jump height at 10’A-FIET, but compared to LDC (MD = 1.74, *P* = 0.001, 95% CI [0.6–2.8]) and PLA (MD = 2.77, *P* = 0.002, 95% CI [0.9–4.6]) vertical jump height of HDC was higher (Figure 4).

Baseline-CMR compared to baseline showed that HDC, LDC, and PLA increased vertical jump height (*P* < 0.05), but no significant changes were observed in CON (*P* > 0.05) (Table 2). Compared to the CMR-baseline, the height of the vertical jump in IA-FIET demonstrated a significant decrease for CON and HDC (*P* < 0.05), but no significant changes were observed in PLA and LDC (*P* > 0.05). The vertical jump height of baseline compared to IA-FIET also indicated that only CON witnessed a significant decrease (*P* < 0.05) and the changes of HDC, LDC, and PLA were not significant (*P* > 0.05). In 5”A-FIET and 10”A-FIET, the height of the vertical jump increased compared to IA-FIET (*P* < 0.05). Also, PLA, LDC, and HDC showed an increase in vertical jump height in 5”A-FIET and 10”A-FIET compared to baseline (*P* < 0.05), while no significant changes were observed in CON (*P* > 0.05). In addition, HDC in 5”A-FIET and 10”A-FIET showed a significant increase in vertical jump height compared to baseline-CMR (*P* < 0.05) (Table 2). LDC, PLA, and CON did not have significant changes in 5”A-FIET and 10”A-FIET compared to baseline-CMR (*P* > 0.05). Also, 10”A-FIET compared to 5”A-FIET had no significant changes in the vertical jump height of PLA, CON, LDC and HDC (*P* > 0.05) (Table 2).

**Table 2.** Comparison of time series of perceived fatigue and vertical jump height in four experimental conditions.

variables	Time 1	Time 2	CON		PLA		LDC		HDC	
			sig	95% CI	sig	95% CI	sig	95% CI	sig	95% CI
Perceived fatigue	Baseline	IA-FIET	0.000	82.7–90.7	0.000	75.9–85.8	0.000	80–87.9	0.000	80.7–86.2
		5’A-FIET	0.000	42.8–56.5	0.000	37.4–48	0.000	39.5–51	0.000	42.9–50.8
		10’A-FIET	0.000	25.2–38.1	0.000	16.9–29.4	0.000	18.7–30.1	0.000	19.4–34
	IA-FIET	5’A-FIET	0.000	31.2–43	0.000	32.5–43.8	0.000	32.3–45	0.000	31.8–41.5
		10’A-FIET	0.000	48–62.3	0.000	50.6–64.8	0.000	52.3–66.9	0.000	48.1–65.6
	5’A-FIET	10’A-FIET	0.000	12.4–23.7	0.000	14.9–24.2	0.000	15.7–26.1	0.000	14.9–25.4
Vertical jump height	Baseline	Baseline-CMR	0.999	0.3–0.5	0.003	0.5–3	0.002	0.7–3.8	0.000	2–4.7
		IA-FIET	0.001	09–4.9	0.999	1.9–3.3	0.999	2.4–2.6	0.999	1.6–3.2
		5’A-FIET	0.999	2.2–2.9	0.007	0.7–5.6	0.000	1.8–7.5	0.000	3.6–7.9
		10’A-FIET	0.999	2–3.5	0.003	1–6.2	0.001	1.4–6.7	0.000	3.8–8.2
	Baseline-CMR	IA-FIET	0.002	0.8–4.8	0.051	0.01–4.8	0.17	0.5–5.2	0.02	0.3–4.9
		5’A-FIET	0.999	2.1–2.8	0.70	0.9–3.6	0.17	0.4–4.8	0.02	0.2–4.4
		10’A-FIET	0.999	0.6–4.5	0.40	0.8–4.5	0.42	0.8–4.6	0.005	0.6–4.5
	IA-FIET	5’A-FIET	0.000	1.9–3.5	0.000	2–5.5	0.000	2.9–6.1	0.000	3.5–6.4
		10’A-FIET	0.000	1.7–5.6	0.000	2.5–6.1	0.000	2.2–6.2	0.000	3–7.4
	5’A-FIET	10’A-FIET	0.17	0.2–2.6	0.999	1.6–2.6	0.999	1.6–2.2	0.999	1.2–1.7

Baseline-CMR: Baseline coffee mouth-rinsing, FIET: Futsal intermittent endurance test, IA-FIET: immediately after the FIET, 5”A-FIET: 5 minutes after the FIET, 10”A-FIET: 10 minutes after the FIET, LDC: low dose coffee. HDC: High dose coffee, PLA: Placebo, CON: Control.

**Figure 5.**
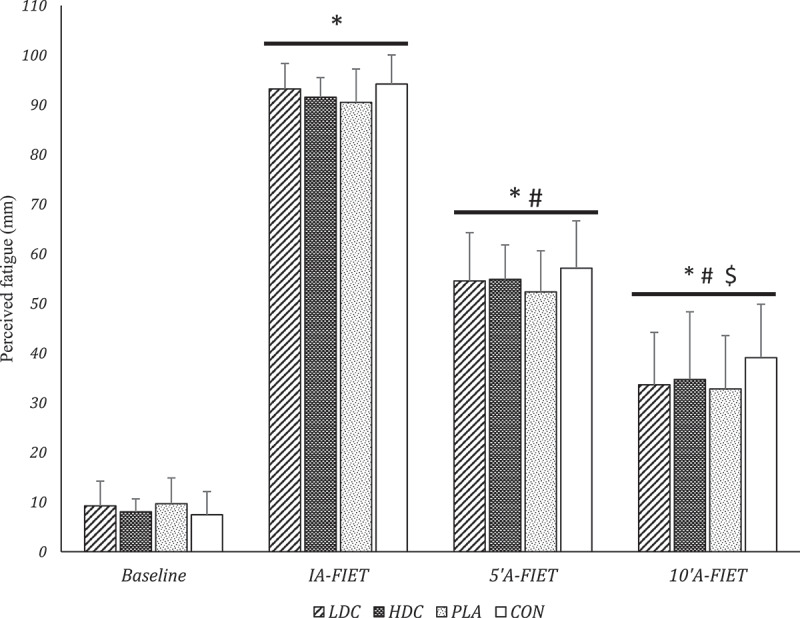
Perceived fatigue (measured by VAS) of participants in different conditions. IA-FIET: immediately after FIET, 5”A-FEIT: 5 min after FIET, 10”AF: 10 min after FIET. LDC: low dose coffee, HDC: High dose coffee, PLA: Placebo, CON: Control. * Significant difference compared to Baseline (P < 0.05), # Significant difference compared to IA-FIET (P < 0.05), $ Significant difference compared to 5’A-FEIT (P < 0.05).

The main effect of time [F = 1911.5, *P* = 0.000, pEta^2^ = 0.98] on perceived fatigue was significant. However, the main effect of condition [F = 2.2, *P* = 0.09, pEta^2^ = 0.09] and the condition × time interaction [F = 1.5, *P* = 0.19, pEta^2^ = 0.06] were not significant. Multiple comparisons showed that the perceived fatigue for all conditions in IA-FIET significantly increased compared to baseline (*P* < 0.05) (Table 2). Compared to IA-FIET, perceived fatigue revealed a significant decrease in 5”A-FIET and 10”A-FIET, however, it was higher than the baseline levels (*P* < 0.05) (Table 2). In addition, perceived fatigue in 10”A-FIET was significantly lower than in 5”A-FIET (*P* < 0.05) (Table 2) and (Figure 5).

## 3. Discussion

The findings of the present study showed that repeated CMR (dose-related) improves specific endurance of futsal and lower limb explosive power (Figures 3 and 4). Also, repeated CMR enhanced lower limb power recovery after exercise-induced fatigue (5”A-FIET and 10”A-FIET). Also, repeated CMR improves the explosive power performance of the lower limb immediately after FIET-induced fatigue and accelerates its recovery.

These findings are consistent with some previous findings. For example, it has been reported that repeated CMR can be effective for increasing the distance covered (30-min cycling time trials/30-min arm crank time trials) [16,20], mean power output in the repeated sprint test (5 × 6 maximal sprints/24s active recovery) [17,26], time to exhaustion in cycling [21], and performance in the Simon task [27]. Also, Van Cutsem et al. (2018) showed that repeated CMR can reduce mental fatigue [28]. In addition, the findings of the present study confirm the suggestions of da Silva et al. (2021) about the possible positive effect of repeated caffeine mouth rinsing on physical performance compared to a single CMR. [18]. However, a study has shown that repeated CMR does not improve time trial endurance performance [29]. Methodological differences as well as the demographic characteristics of the participants can be the cause of the contradictory findings. For example, the exercise test protocol (type, duration, and intensity) in the study of Doering et al. (2014) was different compared to the present study (cycling at 75% of peak aerobic power output for 60 min VS FIET and vertical jump height) [29]. Therefore, it was suggested that future studies pay more attention to the interaction of exercise parameters (intensity, type, and duration) and caffeine/CMR on athletes’ performance.

In intermittent performance such as futsal, the effects of fatigue on performance are shown by reducing the work rate and it has a negative effect on important indicators of the game such as sprints, jumps, and kicks [30]. Also, in futsal competition (as time goes on) the distance covered and the efficiency of kicks decrease [2]. Therefore, the positive effect of repeated CMR on improving the FIET distance covered and vertical jump performance can be functionally important to delay the fatigue of competitive futsal players. In addition, futsal consists of two 20-min periods (with a 15 min half-time) and a time-out can be used in each half, therefore, mouth rinsing with ergogenic substances such as caffeine in these short opportunities to reduce fatigue and increase performance is a practical strategy for futsal players. One of the possible mechanisms of this effect could be that sensing substances in the mouth can quickly affect the brain’s neural function and motor outputs [13,17]. In addition, it has been noted that the oral cavity has bitter taste receptors that are activated by exposure to bitter compounds such as caffeine [13,14]. Therefore, based on the evidence, the positive effect of CMR on performance can be attributed to increasing the activity of oral receptors and in turn increasing the excitability and conductivity of the central nervous system [13,14,17,18,31]. The mechanisms by which mouthwash with bitter-tasting foods such as caffeine/coffee improve physical performance are not yet known, but preliminary evidence focuses on the activation of the sympathetic nervous system and brain regions involved in motor control. For example, previous studies have shown that a bitter solution containing quinine elicited greater physiological responses indicating activation of the sympathetic nervous system than sweet, salty, and sour tastes [32]. Salivary absorption of caffeine can also be another mechanism of performance improvement caused by CMR. There are observations that the absorption of caffeine in the oral cavity is faster compared to the capsule form and leads to a greater ergogenic response [33]. In turn, increasing plasma caffeine can improve performance by directly affecting muscle tissue, such as increasing the release of calcium from the sarcoplasmic reticulum and increasing the activity of the sodium-potassium ATPase enzyme [34–38]. Also, plasma caffeine can reduce muscle pain with its hypoalgesic effect by blocking peripheral and central adenosine A1 and A2 receptors [17,39]. Because the plasma levels of caffeine were not measured in the present study, the role of this mechanism in improving performance indicators cannot be accurately determined. However, the more pronounced performance enhancement observed in the HDC condition compared to the LDC may be due to increased plasma levels of caffeine in addition to the CNS stimulatory effect. Regarding this hypothesis, some findings indicate that very low doses of caffeine (1.5 mg/kg) can lead to improved exercise performance independently of increasing caffeine plasma levels [40], which is consistent with our findings in LDC and PLA compared to the CON condition. Therefore, it is suggested that the effect of CMR dose on the improvement of performance caused by CNS activation should be more closely compared to caffeine plasma levels.

The findings of the present study showed that the vertical jump performance of all four conditions decreased significantly compared to the baseline immediately after the FIET, but the decrease in the vertical jump height in the HDC and LDC conditions was less than the CON (*P* < 0.05). After 5 min of rest (5”A-FIET), the participants’ vertical height jump increased in all conditions (compared to IA-FIET), and the ergogenic effect of CMR on the improvement of a vertical jump compared to the baseline also reappeared (Figure 4). Peripheral fatigue factors such as muscle creatine-phosphate depletion, metabolic acidosis, and stimulation of III/IV sensory afferents can be the reasons for the decrease in vertical jump performance at IA-FIET [41,42]. However, it has been reported that the reduction of inhibitory mechanisms (activation of small-diameter group III/IV afferents) caused by caffeine can be associated with improved performance in subsequent exercises [37]. Therefore, modulation of III/IV sensory afferents due to CMR may be one of the mechanisms that explain the improvement of vertical jump performance in IA-FIET and 5”A-FIET. In addition, some studies have also shown that caffeine reduces the concentration of plasma and muscle interstitium K^+^, which has a beneficial effect on the neuromuscular junction [38]. Indeed, it has been shown that the failure of neuromuscular transmission after fatigue is reduced by caffeine [43]. It has also been indicated that caffeine improves exercise tolerance despite the reduction of phosphocreatine and H^+^ accumulation, which is consistent with the findings of the present study in IA-FIET [43]. Furthermore, it has been illustrated that CMR can improve sports performance in conditions of fasting muscle glycogen depletion [17], which is consistent with the improvement of vertical jump performance at IA-FIET, 5”A-FIET, and 10”A-FIET. However, the findings of this study showed that CMR after 10 min of rest (10”A-FIET) does not have a profound effect on the improvement of performance (vertical jump height) observed during 5”A-FIET (Figure 4). Therefore, a certain range of oral rinsing repetitions/dosage may influence the ergogenic effects of CMR on performance. In addition, it is possible that the sensitivity of CMR-responsive pathways gradually decreases with increasing repeated exposure to caffeine/coffee mouth rinses. This hypothesis is consistent with the higher vertical jump of HDC compared to LDC at 10”A-FIET. Because a higher dose of CMR led to a greater increase in performance than a lower dose, which could be due to greater stimulation of CMR-responsive pathways. Based on this, it is suggested that future studies pay more attention to the interaction of CMR frequency and dose on improving physical/cognitive performance. In addition, considering the significant difference between HDC and LDC in 10”A-FIET and its disappearance in 5”A-FIET and IA-FIET, it is unlikely that the level of fatigue (before the exercise test) affects the main effects of CMR dose on performance. However, none of the central/peripheral fatigue parameters were evaluated in the present study, so it is suggested that future studies investigate the role of CMR on fatigue factors and performance recovery.

It should be mentioned that the results revealed that a placebo can also be effective in improving FIET and vertical jump performance (at baseline CMR, IA-FIET, 5”A-FIET and 10”A-FIET) compared to CON (*P* < 0.05). However, FIET performance in LDC and HDC was significantly better than placebo (*P* < 0.05). Also, vertical jump performance in HDC was better than placebo in baseline-CMR, IA-FIET, 5”A-FIET, and 10”A-FIET (*P* < 0.05). Therefore, the improvement of FIET and vertical jump performance may be partly due to placebo ergogenic effects. Some previous studies have also confirmed that the ergogenic or physiological effect of the placebo can be comparable to caffeine [44–47], but others have not reported a significant effect [48]. Using deceptive interventions, it has also been reported that a placebo enhanced performance when participants were informed that the substance they were consuming was caffeine [46,47]. Some research has also shown that participants who consciously consumed caffeine increased their performance, and it seemed that the belief that they were consuming caffeine was necessary to obtain an ergogenic effect [49,50]. In this study, decaffeinated coffee was used as a placebo, so participants’ knowledge that they were consuming coffee may partially explain the observed placebo effect on performance improvement. Also, participants’ knowledge of the ergogenic properties of caffeine may have influenced the results observed in the placebo condition.

The present study has some limitations, such as the lack of measurement central/peripheral fatigue parameters. In addition, due to financial and time constraints, the authors of this study could not use the counterbalance design method to eliminate the order effect. Therefore, future studies should include the evaluation of these variables to expand on our findings.

## 4. Conclusion

Overall, the present study showed that repeated CMR with low and high doses can be a useful strategy for improving futsal-specific endurance performance. However, higher dose of CMR appears to have more profound effects on performance improvement than lower doses. In addition, the findings of this study indicated that CMR can maintain the performance of the explosive power of the lower body during FIET-induced fatigue and accelerate its recovery. Also, according to the results of the present study, it is possible to give this practical suggestion to futsal players to use CMR in time-outs and half-time to delay fatigue and enhance power and intermittent performance. It is suggested that future studies pay more attention to identifying the underlying mechanisms of CMR’s effect on sports performance.
